# A Bayesian framework for efficient and accurate variant prediction

**DOI:** 10.1371/journal.pone.0203553

**Published:** 2018-09-13

**Authors:** Dajun Qian, Shuwei Li, Yuan Tian, Jacob W. Clifford, Brice A. J. Sarver, Tina Pesaran, Chia-Ling Gau, Aaron M. Elliott, Hsiao-Mei Lu, Mary Helen Black

**Affiliations:** Ambry Genetics, Aliso Viejo, California, United States of America; CNR, ITALY

## Abstract

There is a growing need to develop variant prediction tools capable of assessing a wide spectrum of evidence. We present a Bayesian framework that involves aggregating pathogenicity data across multiple *in silico* scores on a gene-by-gene basis and multiple evidence statistics in both quantitative and qualitative forms, and performs 5-tiered variant classification based on the resulting probability credible interval. When evaluated in 1,161 missense variants, our gene-specific *in silico* model-based meta-predictor yielded an area under the curve (AUC) of 96.0% and outperformed all other *in silico* predictors. Multifactorial model analysis incorporating all available evidence yielded 99.7% AUC, with 22.8% predicted as variants of uncertain significance (VUS). Use of only 3 auto-computed evidence statistics yielded 98.6% AUC with 56.0% predicted as VUS, which represented sufficient accuracy to rapidly assign a significant portion of VUS to clinically meaningful classifications. Collectively, our findings support the use of this framework to conduct large-scale variant prioritization using *in silico* predictors followed by variant prediction and classification with a high degree of predictive accuracy.

## Introduction

The recent surge of sequencing-based clinical genetic testing has put a spotlight on associated challenges in data interpretation. While advances in genomics allow for the development of new genetic tests at an unprecedented pace and complexity, the interpretation of results has remained a largely manual and time-consuming process that is not scalable to the volume and diversity of available data [1]. In particular, the rich evidence in well classified variants is not effectively incorporated in classification schemes relying on manual processing of large-scale information.

The pathogenicity of a genetic variant can be assessed by various evolutionary, functional and structural *in silico* scores and a range of evidence from clinical, family history, co-occurrence and co-segregation data, as well as the published findings of case-control, cohort or family-based studies [2, 3]. One approach to variant classification is to follow a rule-based scoring system, in which each line of evidence is converted into a score and the summary score from all available evidence is used to determine the classification [4–6]. This rule-based approach assumes variants have strong and consistent evidence that is equally applicable across the genome. It also makes use of only qualitative evidence and does not leverage existing knowledge pertaining to other well classified variants. Another approach to variant classification is the multifactorial likelihood method, in which the prior probability of pathogenicity is obtained by using a genome-wide *in silico* (S1 Table) or ensemble (S2 Table) prediction method and the posterior probability is derived by aggregating the prior probability over multiple quantitative evidence [7, 8]. While this multifactorial method is computationally simple, it does not account for gene-specific differences and variability of the estimated prior probability. A third approach uses allele frequencies and multiple *in silico* predictors in a Bayesian logistic regression model that includes priors based on case-control proportions of carriers reported in the literature or public databases, a categorical covariate for gene-specific effects, and fixed model terms for each gene tested [9]. Such models improved prediction accuracy over alternative approaches, but did not allow for all parameters to be freely estimated for each gene or make use of the wide range of available evidence.

To efficiently and accurately determine the pathogenicity of genomic variants, there is a growing need to develop data-driven tools that are capable of assessing a wide array of evidence associated with each variant while leveraging information that is readily available for well classified variants. To address this need, we developed a Bayesian framework for variant prediction that aggregates multiple *in silico* scores and evidence statistics in both quantitative and qualitative forms, and validated the models in genes associated with hereditary cancer syndromes. Our approach improves upon existing methods by leveraging the vast information available for classified variants, quantifying gene-specific *in silico* effects while incorporating both quantitative and qualitative evidence, and predicting the pathogenicity of each variant using a probability distribution to account for uncertainty.

## Results

### Analytical framework

**Fig 1:**
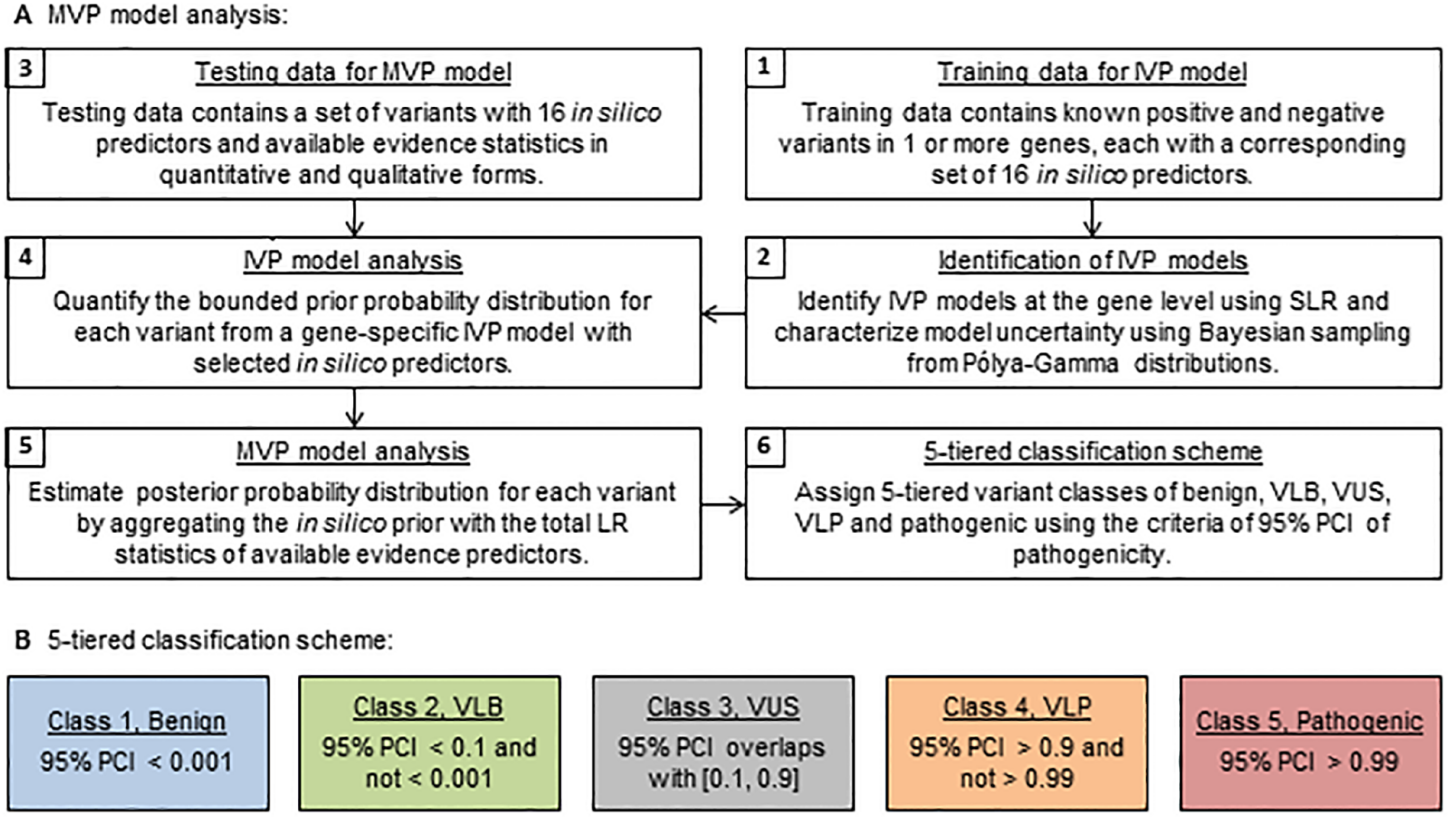
Algorithm modules of multifactorial model analysis for variant prediction and classification. (**A**) Modules of Bayesian multifactorial model analysis for variant prediction and classification. SLR = stepwise logistic regression. (**B**) 5-tiered variant classification scheme based on the estimated 95% probability credible interval (PCI) of variant pathogenicity.

Our Bayesian framework is a data-driven model-based tool for variant prediction and classification analysis (Fig 1). Initially, a set of gene-specific *in silico* predictors is selected from publically available *in silico* scores. An *in silico* variant prediction (IVP) model is used to predict a preliminary level of variant pathogenicity based on a training dataset that contains variants with known classification and their accompanying 16 *in silico* predictors. Next, the prior probability distribution of pathogenicity for each variant is estimated from a corresponding IVP model followed by a rescaled transformation, and the posterior probability distribution is estimated from a multifactorial variant prediction (MVP) model that aggregates the prior distribution over multiple evidence predictors (Fig 1A). Finally, variant classification is assigned to 5-tiered classes of benign, variant of likely benign (VLB), variant of uncertain significance (VUS), variant of likely pathogenic (VLP) and pathogenic based on the probability distribution of pathogenicity (Fig 1B).

### *In silico* model analysis

**Fig 2:**
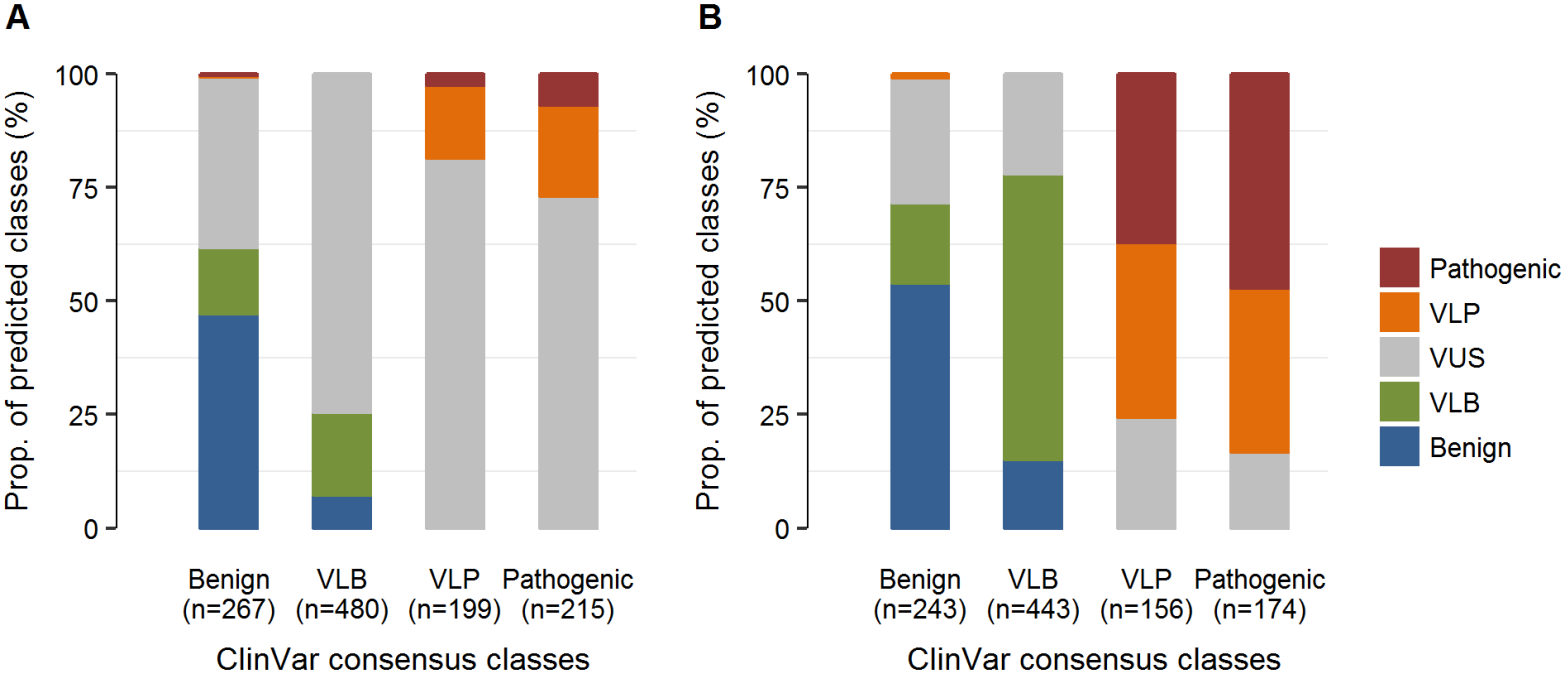
Outcome of 5-tiered predicted classes in MGPT data. (**A**) The proportions of predicted classes from gene-specific IVP model analysis in which each prediction was evaluated from a subset of 16 *in silico* predictors. The analysis was based on 1,161 class known missense variants in 10 genes using LOOCV. (**B**) The proportions of predicted classes from MVP model analysis in which each prediction aggregated a prior distribution from IVP model with the available evidence predictors. The analysis was based on 1,016 variants with any available evidence statistics.

For each of 10 genes that collectively constitute the multigene panel test (MGPT) data containing missense variants with known ClinVar consensus classification outcomes of benign, VLB, VLP and pathogenic, we constructed a training dataset and derived an IVP model that retained 1 to 4 *in silico* predictors from the 16 candidate standalone scores tested (S3 Table). When these gene-specific IVP models were evaluated in all 1,161 class-known variants using a leave-one-out cross validation (LOOCV) method and the 5-tiered classification scheme, 360 (31.0%) were predicted to be concordant with their known classes, 277 (23.9%) were categorized one level above or below their known classes (e.g., benign as VLB, pathogenic as VLP, etc.), 520 (44.8%) were classified as VUSs, and 4 (0.3%) were discordant (e.g., pathogenic/VLP classified as benign/VLB or vice versa) and therefore noted as false negatives or false positives (Fig 2A; S4 Table). Thus, while the IVP model yielded high positive predictive value (PPV), negative predictive value (NPV) and accuracy (100.0%, 99.2% and 99.4%, respectively), sensitivity and specificity were modest (35.7% and 65.5%, respectively) (Table 1, lower section).

**Table 1:** *In silico* variant prediction in MGPT data.

Method^a^	No. by Predicted Outcomes^b^					Performance Statistics^c^						
	TP	TN	FP	FN	VUS	Sen	Spe	PPV	NPV	Acc	AUC	P_VUS_
Standalone predictor:												
MutPred	101	496	1	17	546	0.244	**0.664**	0.990	0.967	0.971	**0.939**	**0.470**
phyloP vertebrate	75	438	6	15	627	0.181	0.586	0.926	0.967	0.961	0.915	0.540
MutationAssessor	71	315	0	9	766	0.171	0.422	**1.000**	0.972	0.977	0.891	0.660
FATHMM	99	276	2	6	778	0.239	0.369	0.980	0.979	0.979	0.884	0.670
AGVGD	217	0	18	0	926	**0.524**	0.000	0.923	NA	0.923	0.878	0.798
Polyphen2 HVAR	0	414	0	21	726	0.000	0.554	NA	0.952	0.952	0.878	0.625
Siphy	0	277	0	5	879	0.000	0.371	NA	**0.982**	**0.982**	0.863	0.757
LRT	0	291	0	8	862	0.000	0.390	NA	0.973	0.973	0.862	0.742
GERP++	0	329	0	12	820	0.000	0.440	NA	0.965	0.965	0.860	0.706
Polyphen2 HDIV	0	404	0	26	731	0.000	0.541	NA	0.940	0.940	0.859	0.630
SIFT	0	315	0	12	834	0.000	0.422	NA	0.963	0.963	0.856	0.718
PROVEAN	50	102	1	2	1,006	0.121	0.137	0.980	0.981	0.981	0.855	0.866
phastCons mammalian	0	361	0	14	786	0.000	0.483	NA	0.963	0.963	0.838	0.677
phastCons vertebrate	0	455	0	10	696	0.000	0.609	NA	0.978	0.978	0.836	0.599
phyloP mammalian	0	213	0	5	943	0.000	0.285	NA	0.977	0.977	0.742	0.812
Grantham	0	0	0	0	1,161	0.000	0.000	NA	NA	NA	0.662	1.000
												
Meta-predictor:												
IVP	148	489	0	4	520	**0.357**	0.655	**1.000**	**0.992**	**0.994**	**0.960**	**0.448**
REVEL	90	475	4	20	572	0.217	0.636	0.957	0.960	0.959	0.942	0.493
MetaSVM	0	518	0	16	627	0.000	**0.693**	NA	0.970	0.970	0.940	0.540
Eigen	22	497	0	4	638	0.053	0.665	**1.000**	**0.992**	0.992	0.932	0.550
Eigen PC	12	506	0	5	638	0.029	0.677	**1.000**	0.990	0.990	0.927	0.550
CADD	61	330	17	3	750	0.147	0.442	0.782	0.991	0.951	0.906	0.646
MutationTaster	0	517	0	13	631	0.000	0.692	NA	0.975	0.975	0.894	0.543

^a^The *in silico* variant prediction analyses were evaluated from gene-specific prediction models from each of 16 standalone predictors and 7 meta-predictors, respectively, in which the IVP predictor was derived from the 16 standalone predictors. Each analysis was evaluated in MGPT data containing 1,161 missense variants (747 negatives, 414 positives) using LOOCV. Results are listed in descending order of AUC values among models using standalone predictors and meta-predictors, respectively.

^b^Predicted outcomes were derived from the predicted positive/negative categories and the known ClinVar consensus classes. TN = true negative, TP = true positive, FN = false negative, FP = false positive.

^c^Performance statistics were reported as Sen = sensitivity, Spe = specificity, PPV, NPV, Acc = accuracy, AUC, and P_VUS_ = proportion of variants classified as VUS. NA = not able to calculate. The best performance statistics among comparison *in silico* prediction methods are highlighted in bold.

**Fig 3:**
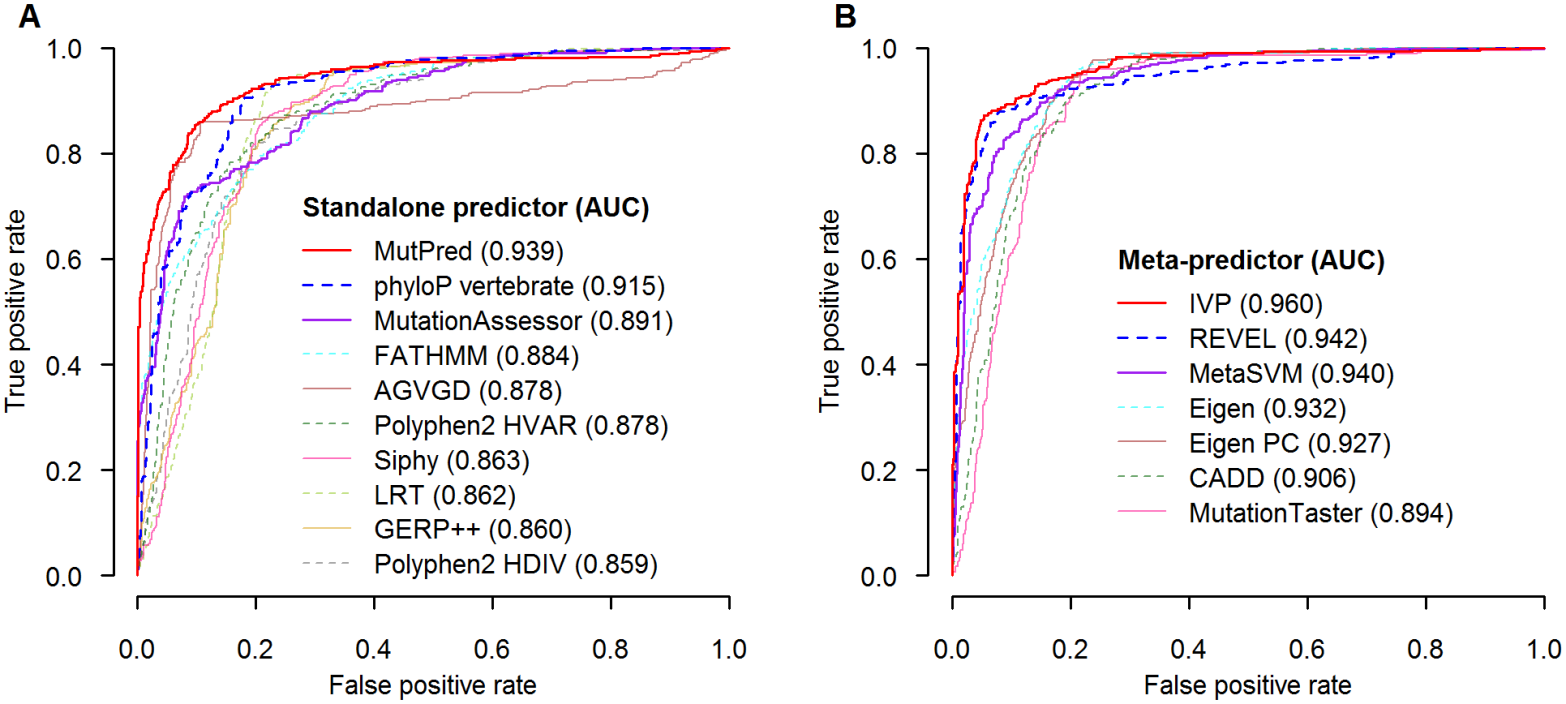
Comparison of AUC statistics of standalone and meta *in silico* predictors in MGPT data. (**A**) AUC statistics of top 10 standalone *in silico* predictors. (**B**) AUC statistics of 7 meta *in silico* predictors. The analysis models in legend were listed in descending order of AUC values. Abbreviations: AUC = area under the receiver operating characteristic curve; IVP = *in silico* variant prediction; MGPT = multigene panel test.

We next compared the predictive performance of the IVP model to that of each individual *in silico* predictor. Using the same 1,161 class-known variants, the gene-specific IVP models had the highest sensitivity (35.7%), PPV (100.0%), NPV (99.2%), accuracy (99.4%), and AUC (96.0%), and lowest proportion of VUS (44.8%) compared to each of the 16 standalone and 6 meta-predictor models (Table 1). Among all 23 *in silico* models, the AUC statistics were highest for IVP model and followed by REVEL and MetaSVM (Fig 3A and 3B; Table 1: AUC_IVP_ = 0.960 vs. AUC_REVEL_ = 0.942, p = 0.01; AUC_IVP_ = 0.960 vs. AUC_MetaSVM_ = 0.940, p = 0.002).

### Multifactorial model analysis

Among the 1,161 variants included in this analysis, 1,016 (87.5%) had data available for at least one evidence statistic from qualitative and/or quantitative sources. When applying our MVP model analysis to these variants, predictive performance improved as expected when compared to that of the IVP models (Table 2, upper section vs. lower section). The proportions of variants classified as concordant with their known classes, categorized one level above or below their known classes, as VUSs, and discordant between benign/VLB and pathogenic/VLP were 551 (54.2%), 231 (22.7%), 232 (22.8%) and 2 (0.2%), respectively (Fig 2B and S4 Table). With 2 false classifications and 782 appropriately categorized as benign/VLB or pathogenic/VLP variants, the MVP model analysis achieved 99.2% PPV (95% CI: 97.1% or above) and 100.0% NPV (95% CI: 99.1% or above). Moreover, in subsets of variants with moderate evidence (total likelihood ratio (LR) statistic <0.1 or >10) or strong evidence (total LR statistic <0.01 or >100) evidence for benign or pathogenic, 95.0% (568/598) and 100% (297/297) were correctly classified into the clinically informative classes of benign/VLB and pathogenic/VLP, respectively (Table 2, upper section). In particular, for the subset of variants with total LR statistic <0.01 or >100, we observed ideal performance statistics: 100.0% PPV, 100% NPV, 100% AUC, and 0% predicted VUS (Table 2, upper section).

**Table 2:** Multifactorial variant prediction in MGPT data.

Method and Data (n_-_, n_+_)^a^	No. by Predicted Outcomes^a^					Performance Statistics^a^						
	TP	TN	FP	FN	VUS	Sen	Spe	PPV	NPV	Acc	AUC	P_VUS_
Using all evidence data:												
Any evidence data (686, 330)	263	519	2	0	232	0.797	0.757	0.992	1.000	0.997	0.998	0.228
LR < 0.1 or > 10 (372, 226)	210	358	2	0	28	0.929	0.962	0.991	1.000	0.996	0.999	0.047
LR < 0.01 or > 100 (223, 74)	74	223	0	0	0	1.000	1.000	1.000	1.000	1.000	1.000	0.000
LR < 0.001 or > 1,000 (155, 23)	23	155	0	0	0	1.000	1.000	1.000	1.000	1.000	1.000	0.000
												
Using only auto-computed evidence:												
Any evidence data (618, 255)	95	287	2	0	489	0.373	0.464	0.979	1.000	0.995	0.986	0.560
LR < 0.1 or > 10 (225, 35)	31	212	2	0	15	0.886	0.942	0.939	1.000	0.992	0.996	0.058
LR < 0.01 or > 100 (174, 8)	8	174	0	0	0	1.000	1.000	1.000	1.000	1.000	1.000	0.000
LR < 0.001 or > 1,000 (107, 0)	0	107	0	0	0	NA	1.000	NA	1.000	1.000	NA	0.000

^a^Multifactorial variant predictions by MVP model analyses were conducted using either all available evidence data, which includes all quantitative and qualitative evidence predictors (total variants = 1,016), or only the 3 auto-computed predictors from readily available databases: family history, co-occurrence and mutation hotspot indicator (total variants = 873), respectively. The prior model for the MVP analysis was constructed for each variant using LOOCV. The n_-_ and n_+_ values refer to the numbers of negative and positive variants, respectively, in the analytical dataset. LR = total LR from all evidence statistics or auto-computed ones. The abbreviation terms of predicted outcomes and performance statistics are same as those in footnotes b and c of Table 1.

Given that some evidence may lend itself to automated computation, while others may require manual examination or adjustment [10], we also applied MVP model analysis using a subset of auto-computed predictors for which data were readily available: co-occurrence, family history and mutation hotspot. Among the 1,161 variants initially included, 873 (75.2%) had data for at least one of these 3 predictors. Results of the constrained MVP model for these variants were highly accurate, with 98.6% AUC (Table 2, lower section); the proportions of variants classified concordant with their known classes, categorized one level above or below their known classes, as VUSs, and discordant between benign/VLB and pathogenic/VLP were 260 (29.8%), 122 (14.0%), 489 (56.0%) and 2 (0.2%), respectively (Table 2, lower section and S4 Table). Overall, 32.9% (382/1,161) of variants were appropriately categorized as benign/VLB or pathogenic/VLP. Among variants with strong evidence for a benign or pathogenic classification (total LR statistic <0.01 or >100, respectively), 100.0% (182/182) of variants were correctly classified as benign/VLB and pathogenic/VLP (Table 2, lower section).

## Discussion

We present a Bayesian framework consisting of IVP models that assess variant pathogenicity using a subset of gene-specific *in silico* predictors, and an MVP model that aggregates this result with information from a variety of qualitative and quantitative evidence sources to accurately and robustly predict the pathogenicity classes of missense variants. The performance of MVP model analysis demonstrates that this approach is capable of leveraging a vast constellation of pathogenicity information available in large-volume data, and has important implications for increased clinical utility given its high predictive accuracy, which was cross validated in over 1,000 classified missense variants.

The IVP and MVP model analyses have several distinct features that allow for improved prediction accuracy and practical utility. First, IVP model prediction is conducted with data-driven model terms derived from gene-specific training data, and is supported by a data expansion procedure to accommodate a sparse data scenario in which the training data contains an insufficient number of class-known variants. Notably, our IVP model quantifies the pathogenicity of each variant with a probability distribution, instead of a probability value, which allows for improved prediction robustness and accuracy by accounting for estimation uncertainty. Second, an exact and fast Bayesian sampling procedure using data-independent Pólya-Gamma distributions is adapted for model estimation [11, 12], which facilitates analysis without the estimation of data-dependent priors on a gene-by-gene basis. Third, the incorporation of evidence statistics in qualitative forms, which are typically collected for rule-based classification schemes, makes the MVP model analysis capable of incorporating broader types of evidence statistics for improved practical utility, although the MVP model is not limited by the use of these data. We demonstrated that MVP model performance is highly accurate even when only three auto-computed quantitative evidence predictors are included in the model. Lastly, the use of 95% PCI is designed to reduce the potential misclassification between benign/VLB and pathogenic/VLP by accounting for the variability in the predicted probability of variant classification.

When applied to the 10 genes with varying numbers of class-known missense variants, the gene-specific IVP models outperformed 16 standalone and 6 meta-predictors each based on a genome-wide *in silico* score. These results support the notion that universal prediction models tend to have varied performance across different genes, and gene-specific classifiers incorporating phenotype data and established disease-causing evidence can improve prediction accuracy [13–15]. For multifactorial variant classification, the MVP model correctly assigned 77% of variants to its precise or closely matched class and misclassified only 0.2% of variants between benign/VLB and pathogenic/VLP. In addition, we show that using auto-computed evidence statistics derived from commonly collected and readily available phenotype and genotype data, 33% of evaluated variants can be correctly classified to their precise or closely matched classes. These results highlight the practical utility of applying IVP model analysis for large-scale variant prioritization and MVP model analysis for variant classification.

Despite the fact that gene-specific models outperform those based on genome-wide information, IVP models can be unstable when the gene-specific training data is of small sample size and/or contains many ambiguously classified variants. Other approaches, such as a weighted average of gene-specific and panel-specific models might improve model robustness and prediction accuracy, and remain to be investigated. The IVP model presented here is limited to continuous *in silico* predictors, whereas categorical features such as domain effects and variant types may also be informative [9]. Availability of evidence statistics is also a limiting aspect of MVP model analysis; although the Bayesian MVP model we presented classified a large proportion of variants with a high degree of accuracy, the remaining VUS were due to either no (145/1,161 = 12.5%) or insufficient (232/1,161 = 20.0%) evidence statistics. In addition, models that account for correlations among evidence statistics [16], incorporate interaction and non-linear effects [9], and/or integrate LR statistics using distribution-based sampling approaches have the potential for improved variant prediction and classification.

Our Bayesian IVP and MVP model analyses form a data-driven framework for variant prediction and classification in aggregating a broad spectrum of pathogenic information. These model-based approaches are adaptive to the complexity of large-scale data, and are applicable to a wide variety of genes and phenotype conditions, provided that suitable training data are available. Importantly, these models afford an opportunity to accurately and efficiently reclassify VUS, and as such have the potential to improve the information on which clinical decisions are based.

## Methods

### Data

To assess prediction performance, we obtained 1,161 classified missense variants identified in 10 MGPT genes (*BRCA1*, *BRCA2*, *CDH1*, *PALB2*, *PTEN*, *TP53*, *MLH1*, *MSH2*, *MSH6* and *PMS2*) from ClinVar (https://www.ncbi.nlm.nih.gov/clinvar/, downloaded in April 2017), compiled *in silico* predictor information from dbNSFP (https://sites.google.com/site/jpopgen/dbNSFP, downloaded in June 2017) and AGVGD (http://agvgd.hci.utah.edu/, released Sept 2014) databases, and collected variant-specific evidence statistics from Ambry Genetics databases, respectively (S1 Data: MGPT data with all relevant variables). Variants obtained from ClinVar were those with classifications established by expert panel review or deposited by any of the 6 submitters that consistently provide assertion criteria: Ambry Genetics, Emory, GeneDx, InSiGHT, InVitae and SCRP. For each variant, we defined its consensus class per the following hierarchy: 1) we selected the most supported category among negative, positive and VUS, where negative and positive are designated as benign/VLB and pathogenic/VLP, respectively (i.e., positive if *N*_*positive*_ > max(*N*_*negative*_, *N*_*VUS*_) or negative if *N*_*negative*_ > max(*N*_*positive*_, *N*_*VUS*_); and 2) if the number of submitters is tied, and there was no conflict between positive and negative classes, we assigned positive or negative as appropriate (i.e., positive if *N*_*positive*_ = *N*_*VUS*_ and *N*_*negative*_ = 0 or negative if *N*_*negative*_ = *N*_*VUS*_ and *N*_*positive*_ = 0). Variants with conflicting classes (i.e., *N*_*positive*_ = *N*_*negative*_), or classified as VUS by all submitters, were excluded from analysis. The number of variants with ClinVar consensus classes varied from 22 to 385 per gene (S5 Table, upper section).

### Predictors and their derivation

The dataset used for IVP model training contained a set of class-known variants which are designated benign, VLB, VLP and pathogenic. The response variable *y* is a binary outcome for pathogenicity, with 1 for pathogenic or VLP and 0 for benign or VLB.

The predictor variables for IVP model analysis included 16 individual *in silico* scores from publically available variant prediction tools (i.e., Grantham [17], GERP++ [18], phastCons (for vertebrate and mammalian genomes) [19], AGVGD [20], SIFT [21], MutPred [22], SiPhy [23], LRT [24], phyloP (for vertebrate and mammalian genomes) [25], Polyphen2 (built on HumVar and HumDiv datasets) [26], MutationAssessor [27], PROVEAN [28] and FATHMM [29]; description in S1 Table). Six meta *in silico* scores were collected for comparisons of prediction performance (i.e., MutationTaster [30], CADD [31], REVEL [32], Eigen (for overall and eigendecomposition) [33] and MetaSVM [34]; description in S2 Table). Missing values of *in silico* predictors were imputed with the *k*-nearest neighbors method implemented in R package DMwR2 version 0.02. The missing values for a given variant were assigned the average value of the non-missing values for that predictor from its *k* = 40 nearest neighboring variants. *In silico* predictors with non-unit scale values (i.e., Grantham, GERP++, AGVGD, SiPhy, phyloP, MutationAssessor, PROVEAN, FATHMM, CADD, Eigen and MetaSVM) were transformed to unit scale of 0 to 1 by (*x*_*raw*_ − *x*_*min*_)/(*x*_*max*_ − *x*_*min*_), where *x*_*raw*_ is the *in silico* score in its original scale and *x*_*min*_ and *x*_*max*_ are the minimum and maximum score values, respectively.

The predictor variables for MVP model analysis included 7 evidence predictors quantified as LR statistics for a) frequency and association, b) co-occurrence, c) co-segregation, d) family history, e) functional evidence, f) structural evidence, and g) other supporting data, respectively (description in S6 Table). For each of the 7 evidence predictors, we extracted qualitative evidence from variant-level classification records characterized by two parameters of *p*_*cut*_ and *p*_*frac*_. Here *p*_*cut*_ is a threshold probability of 0.001, 0.1, 0.9 or 0.99 for assigning a variant to a targeted class of benign, VLB, VLP or pathogenic, respectively, as recommended in the American College of Medical Genetics and Genomics (ACMG) guidelines [2]. Also, *p*_*frac*_ is a fraction of evidence needed to reach a targeted class (e.g., 1, 0.5 and 0.25 are example values for evidence predictors in qualitative form; see S6 Table for numerical illustration). Applying the Bayes rule equation under a null probability of *p*_*null*_ = 0.5, the LR statistic for qualitative pathogenicity evidence is

$$LR(p_{cut},\;p_{frac})={\left(p_{cut}/\left(1-p_{cut}\right)\right)}^{p_{frac}}.$$ (1)

For auto-computed predictors, we derived LR statistics directly from real-time in-house subject-level genotype and phenotype data. The LR statistic of co-occurrence evidence was estimated from a binomial likelihood model [35] under the rationale that a pathogenic variant is less likely to co-occur with a known pathogenic mutation in trans. The LR statistic of family history evidence was derived from a history weighting score model [36] based on the premise that pathogenic mutations are more likely to occur in high-risk individuals while the presence of benign variants is unrelated to personal and family history. The mutation hotspot evidence was filtered out for the existence of at least one pathogenic variant at the same amino acid residue. For evidence statistics derived from two sources, denoted *LR*_1_ and *LR*_2_, the combined evidence of two correlated LR statistics is quantified as

$$LR\left(LR_{1},LR_{2}\right)=\{\begin{array}{ll} \text{max}\left(LR_{1},LR_{2}\right) & if\;both>1,\\ \text{min}\left(LR_{1},LR_{2}\right) & if\;both<1,\\ LR_{1}\times LR_{2} & otherwise. \end{array}$$ (2)

Thus, the available pathogenicity evidence for each variant was summarized into 7 LR statistics, where LR statistic was set to1 for each missing evidence predictor.

### Training data for IVP model

To build a gene-specific IVP model for gene *G*, we constructed a training data A containing all class-known variants in that gene under a minimal sample size requirement of *n*_*negative*_ ≥ 5 and *n*_*positive*_ ≥ 5. Here we define *n*_*negative*_ = *n*_*benign*_ + 0.5× *n*_*VLB*_ and *n*_*positive*_ = *n*_*pathogenic*_ + 0.5× *n*_*VLP*_, where each n_*_ represents the number of class * variants in training data A. The variant classes of benign, VLB, VLP and pathogenic are based on ClinVar consensus classifications, as previously described. For a sparse data scenario in which the variants in training data A satisfy *n*_*negative*_ + *n*_*positive*_ ≥ 5 and either *n*_*negative*_ < 5 or *n*_*positive*_ < 5, we implemented a data expansion procedure based on the assumption that variants with similar *in silico* scores residing in two genes known to influence the same phenotype should have a more similar degree of pathogenicity than those from two randomly selected genes. This procedure resulted in a training data A for gene *G* that includes additional variants from other similar genes, and was implemented as follows:

1. Computed the mean distance for all variant pairs between gene *G* and each other gene *H* ⊂ G*, where G* is a gene set for all genes from the same panel or pathway except gene *G*. The distance between variants *g* ∈ *G* and *h* ∈ *H* was defined as the Euclidean distance of *d*_*gh*_ = *sqrt*(Σ_*i* = 1,⋯,*I*_(*x*_*gi*_−*x*_*hi*_)^2^), where vectors (*x*_*g*1_,⋯,*x*_*gI*_) and (*x*_*h*1_,⋯,*x*_*hI*_) are the *I* = 16 unit scaled individual *in silico* predictor values of variants *g* and *h*, respectively.
2. Chose a most similar gene *G’* ⊂ G* that showed the minimal mean distance with analysis gene *G* and merged the negative and/or positive variants one by one from *G’* to A in descending order of between-variant distance until both *n*_*negative*_ ≥ 5 and *n*_*positive*_ ≥ 5 were met or all variants in *G’* were merged into the training data A.
3. If *n*_*negative*_ < 5 or *n*_*positive*_ < 5 remains true, repeated step (2) by choosing another gene *G”* ⊂ (G* − *G’*) and merging variants from *G”* to A until *n*_*negative*_ ≥ 5 and *n*_*positive*_ ≥ 5 were met.

Thus, this data expansion procedure provided a means by which gene-specific training data may be constructed for genes that contain only a few classified variants.

### Identification of IVP model

We derived a gene-specific IVP model by selecting a subset of *in silico* predictors using step-wise logistic regression (SLR) and quantified the probability distribution of pathogenicity using Bayesian logistic regression with coefficients sampled from data-independent Pólya-Gamma distributions. The set of predictors yielding the minimal cross validation error rate among penalty coefficients (ranging from 2 to 8) were retained. The derived IVP model took the form

$$logit(y)=b_{0}+b_{1}z_{1}+\cdots +b_{K}z_{K},$$ (3)

where *Z* = (*z*_1_, …, *z*_*K*_) is a subset of gene-specific *in silico* predictors retained from SLR analysis, *B* = (*b*_0_, *b*_1_, …, *b*_*K*_) are regression coefficients for intercept and slopes, and *logit*(*y*) is the logit transformation of response variable *y* for negative and positive variants. The distributions of regression coefficients *B* of an IVP model were estimated by Bayesian Markov chain Monte Carlo updated from Pólya-Gamma distributions [11], as implemented by the logit function in R package BayesLogit version 0.5.1. For variant prediction purposes, the distributions of *K* + 1 regression coefficients, denoted $B=(b_{kn},0\leq k\leq K,1\leq n\leq N)$, are estimated jointly using *N* = 1,000 Gibbs samples after a burn-in period of 20,000 samples. Thus, the IVP probability distribution of each variant was estimated by its logistic transformation, denoted $\dot{P}=({\dot{p}}_{n},\;1\leq n\leq N|{\dot{p}}_{n}={logit}^{-1}(b_{0n}+\sum_{k}b_{kn}x_{kn}))$.

### Estimation of IVP prior probability distribution

As a principle of multifactorial variant classification analysis, at least two lines of evidence are required for assigning a variant class to benign, VLB, VLP or pathogenic (i.e., classification based on the probability distribution from *in silico* analysis alone is not possible) [2]. To accommodate this framework, the IVP prior distribution of each variant was derived from a rescaled IVP probability distribution in the range of 0.1 to 0.9 with standard deviation proportional to the expected value. Specifically, the IVP prior distribution of a variant, denoted $P=(p_{n},1\leq n\leq N)$, was quantified by a 2-sided shifted probability function

$$p_{n}={\ddot{p}}_{med}+({\ddot{p}}_{n}-{\ddot{p}}_{med})\times sd({\ddot{p}}_{med})/sd({\dot{p}}_{med}),$$ (4)

where $\ddot{P}=({\ddot{p}}_{n},\;1\leq n\leq N|{\ddot{p}}_{n}=0.8\times {\dot{p}}_{n}+0.1)$ is a linearly shifted probability distribution in range of 0.1 to 0.9, ${\dot{p}}_{med}$ and ${\ddot{p}}_{med}$ are the medians of IVP distribution $\dot{P}$ and its shifted distribution $\ddot{P}$, respectively, and $sd({\dot{p}}_{med})=sqrt({\dot{p}}_{med}\times (1-{\dot{p}}_{med}))$ and $sd({\ddot{p}}_{med})=sqrt({\ddot{p}}_{med}\times (1-{\ddot{p}}_{med}))$ are the expected standard deviations of ${\dot{p}}_{med}$ and ${\ddot{p}}_{med}$, respectively. The use of a rescaled prior distribution, as quantified by Eq (4), ensures the variability of IVP distribution is maintained in the prior distribution.

### MVP model analysis

For each variant, the Bayesian MVP model analysis employed a distribution-based formation of Bayes rule to quantify the posterior probability distribution of pathogenicity. Such a posterior distribution, denoted $Q=\{q_{n},1\leq n\leq N\}$, was computed by aggregating the prior probability distribution P from an IVP model analysis and the available LR statistics from 7 evidence predictors through the equation:

$$q_{n}=p_{n}{LR}_{total}/\left(1-p_{n}+p_{n}{LR}_{total}\right).$$ (5)

where *p*_*n*_ is a sample value from the prior distribution, and *LR*_*total*_ = *LR*_*FAA*_ × *LR*_*COC*_ × *LR*_*CSG*_ × *LR*_*FHX*_ × *LR*_*FUN*_ × *LR*_*STR*_ × *LR*_*OTH*_ is the total LR statistic calculated under the assumption that the 7 evidence predictors are statistically independent (see S6 Table for definition of each LR statistic).

### 5-tiered variant classification scheme

We employed a 5-tiered variant classification scheme to assign the predicted classes of benign, VLB, VUS, VLP and pathogenic at the targeted probability thresholds of 0.001, 0.1, 0.9 and 0.99, respectively, following the ACMG guideline (Fig 1A) [2, 37]. The predicted class of each variant was assigned based on the 95% probability credible interval (PCI) of pathogenicity obtained from an IVP model or MVP model (Fig 1B). Here the 95% PCI is defined as the 2-sided 95% range of a probability distribution estimated from a corresponding prediction model using Bayesian sampling from Pólya-Gamma distributions [11]. The use of 95% PCI, instead of a point estimate of probability value, was designed to control the occurrence of false events by accounting for the variability of probability distribution.

### Performance evaluation

To evaluate the performance of variant prediction, we assessed predicted outcomes including true positives (TP; predicted pathogenic/VLP concordant with known class), true negatives (TN; predicted benign/VLB concordant with known class), false positives (FP; benign/VLB predicted to be pathogenic/VLP), and false negatives (FN; pathogenic/VLP predicted to be benign/VLB). Performance statistics such as sensitivity, specificity, PPV, NPV, accuracy, AUC and proportion of predicted variants of uncertain significance (P_VUS_) were assessed [38] (S7 Table). All performance statistics of IVP and MVP model analyses were evaluated using LOOCV to control model overfitting. The 95% confidence interval (CI) of a proportion was estimated from binomial distribution. Comparison of AUC estimates for different methods was performed using Delong’s test [39]. All analyses were conducted with R for Statistical Computing version 3.3.3.

## Supporting information

S1 Table Standalone *in silico* predictors. ^a^ The 16 standalone predictors in this table will be used for building the *in silico* variant prediction (IVP) models.
(DOCX)

S2 Table Meta *in silico* predictors. ^a^ The 6 meta predictors in this table will be used to evaluate the prediction performance.
(DOCX)

S3 Table *In silico* predictors retained in gene-specific IVP models. ^a^ No. of variants in gene-specific training data included the classified variants in MGPT data from the same gene and those from other genes with minimal average distance to meet the sample size criterion of *n*_*neg*_ ≥ 5 and *n*_*pos*_ ≥ 5. ^b^ The IVP models were built on a subset of 16 standardized scores (gra, …, mua) in unit range of 0 to 1 from their original scores (Grantham, …, FATHMM), as follows: gra = (Grantham– 5)/215, ger = (GERP++ + 12.3)/18.5, pcv = phastCons_vertebrate, pcm = phastCons_mannalian, agv = AGVGD/65, sif = 1 –SIFT, mup = MutPred, sip = Siphy/38, lrt = LRT, ppv = (phyloP_vertebrate + 20)/30, ppm = (phyloP_mammalian + 13.3)/14.5, pov = Polyphen2_HVAR, pod = Polyphen2_HDIV, mua = (MutationAssessor + 5.2)/11.7, pro = (PROVEAN + 14)/28, and fat = (FATHMM + 16.2)/26.9.
(DOCX)

S4 Table Cross validation for variant classification in MGPT data. ^a^ IVP model analysis was conducted with 1,161 missense variants. MVP model analyses were evaluated in 1,016 variants with any available evidence and 873 variants with only auto-computed evidence. Total numbers of variants for MVP model analysis were reduced due to some variants did not have the required evidence statistics. Known classes were based on ClinVar consensus classification outcomes, and the predicted classes were evaluated by IVP and MVP models, respectively, using LOOCV.
(DOCX)

S5 Table Number of variants according to ClinVar consensus classification. (DOCX)

S6 Table Evidence items and their likelihood ratio statistics. ^a^ The description of each evidence item follows the Ambry's Variant Classification Scheme. ^b^ All evidence items are summarized into 7 categories for MVP model analysis: FAA: Frequency and association evidence from control populations and case-control studies; COC: Co-occurrence evidence with another mutation; CSG: Co-segregation evidence with disease; FHX: Evidence of personal and family history, *de novo* alternation in family and established diagnosis without other mutation; FUN: Evidence of functional validation or genomic features (includes mutational hotspot); STR: Structural evidence; OTH: Other supporting evidence not included in other evidence groups. ^c^ The effect of each evidence item is graded into 7 levels of P-1, P-4, LP-1, LP-4, LB-1, LB-2 and B-1, respectively. These effect levels are quantified using following criteria under the assumption of no prior knowledge of variant pathogenicity: One evidence item of effect level P-1, or 4 evidence items of effect level P-4, are required to classify a variant to pathogenic; One evidence item of effect level LP-1, or 4 evidence items of effect level LP-4, are required to classify a variant to VLP; One evidence item of effect level LB-1, or 2 evidence items of effect level LB-2, are required to classify a variant to VLB; One evidence item of effect level B-1 is required to classify a variant to benign. ^d^ The effect of each qualitative evidence is transferred to a LR statistic under a null probability of 0.5 (see Eq (1) in Methods section). Numerically, the LR statistics for different evidence effects are calculated as follows: LR(P-1) = 0.99/(1–0.99) = 99; LR(P-4) = 99^0.25^ = 3.1543; LR(LP-1) = 0.95/(1–0.95) = 19; LR(LP-4) = 19^0.25^ = 2.0878; LR(LB-1) = 0.05/(1–0.05) = 0.0526; LR(LB-2) = (1/19)^0.5^ = 0.2294; LR(B-1) = 0.001/(1–0.001) = 0.0010.
(DOCX)

S7 Table Performance statistics for variant classification. ^a^ The number of “total positive variants evaluated” includes true positives (TP), false negatives (FN) and those positives predicted as variants of uncertain significance (VUS). The number of “total negative variants evaluated” includes true negatives (TN), false positives (FP) and those negatives predicted as VUS.
(DOCX)

S1 Data MGPT dataset. The 1,161 missense variants in 10 genes for performance evaluation of *in silico* and multifactorial model analyses reported in this article.
(XLSX)
