# Supplementary material for: A Bayesian framework for efficient and accurate variant prediction
Source: PLoS One. 2018 Sep 13;13(9):e0203553. doi: 10.1371/journal.pone.0203553 (PMC6136750; doi:10.1371/journal.pone.0203553)
Supplement: S1 Table — a The 16 standalone predictors in this table will be used for building the in silico variant prediction (IVP) models. (DOCX) [file pone.0203553.s001.docx]

**S1 Table. Standalone *in silico* predictors**

| **Predictor^a^** | **Method Description** | **Website and Reference** |
| --- | --- | --- |
| Grantham | Grantham score predicts the effect of substitutions between amino acids based on chemical properties. A higher Grantham score reflects a greater evolutionary distance and is considered more deleterious. The score ranges from 5 to 215. | <http://science.sciencemag.org/content/185/4154/862>; Grantham, 1974 [[1](#_ENREF_1)] |
| GERP++ | GERP++ quantifies the evolutionary constraint on sequence regions by identifying regions from sequence alignments depleted of variation due to selection. Positive and negative scores indicate depletion of variation and neutral evolution, respectively. The score ranges from -12.3 to 6.2. | <http://mendel.stanford.edu/SidowLab/downloads/gerp/>; Cooper, et al., 2005 [[2](#_ENREF_2)] |
| phastCons vertebrate  phaseCons mammalian | PhastCons measures the probability that each nucleotide belongs to a conserved element based on multiple alignment and phylogenetic hidden Markov model. The scores for vertebrate and mammalian genomes each ranges from 0 to 1. | <http://genome.ucsc.edu/goldenPath/help/phastCons.html>; Siepel, et al., 2005 [[3](#_ENREF_3)] |
| AGVGD | AGVGD program combines the biophysical characteristics of amino acids and protein multiple sequence alignments to predict where missense substitutions in genes of interest fall in a spectrum from enriched deleterious to enriched neutral. The score ranges from 0 to 65. | <http://agvgd.hci.utah.edu/>; [Tavtigian, et al., 2006](#_ENREF_18) [[4](#_ENREF_4)] |
| SIFT | SIFT predicts whether an amino acid substitution affects protein function based on the degree of conservation of amino acid residues in sequence alignments derived from closely related sequences, collected through PSI-BLAST. The score ranges from 0 to 1 with a smaller value representing a stronger damaging effect. | <http://sift.jcvi.org/>; [Kumar, et al., 2009](#_ENREF_11) [[5](#_ENREF_5)] |
| MutPred | MutPred predicts the functional effects of protein missense mutations by using SIFT along with the gain/loss of 14 different structural and functional features. The score ranges from 0 to 1. | <http://mutpred1.mutdb.org/about.html>; [Li, et al., 2009](#_ENREF_12) [[6](#_ENREF_6)] |
| SiPhy | SiPhy measures conservation from multiple sequence alignment that is due to selection. The score is the log odds ratio of fitted versus neutral likelihoods or the sum of these scores across windows containing the variant. The score ranges from 0 to 38. | <http://portals.broadinstitute.org/genome_bio/siphy/index.html>; [Garber, et al., 2009](#_ENREF_5) [[7](#_ENREF_7)] |
| LRT | LRT computes the two-sided p-value of likelihood ratio test between an evolutionary model with selection versus a neutral evolution model. The score ranges from 0 to 1 with a smaller value representing more deleterious or neutral effect depending on an estimated nonsynonymous-to-synonymous rate ratio. | [Chun and Fay, 2009](#_ENREF_3) [[8](#_ENREF_8)] |
| phyloP vertebrate  phyloP mammalian | PhyloP measures conservation scoring based on multiple alignments of 100 vertebrate genomes and 20 mammalian genomes to human genomes. The scores for vertebrate and mammalian genomes range from -20.0 to 10.0 and -13.3 to 1.2, respectively. These scores may differ by chromosome and human genome assembly. | <http://hgdownload.cse.ucsc.edu/goldenPath/hg19/phyloP100way/>; [Pollard, et al., 2010](#_ENREF_13) [[9](#_ENREF_9)] |
| Polyphen2 HVAR  Polyphen2 HDIV | PolyPhen2 is a tool which predicts possible impact of an amino acid substitution on the structure and function of a human protein using straightforward physical and comparative considerations. The scores from HVAR and HDIV data each ranges from 0 to 1. | <http://genetics.bwh.harvard.edu/pph2/>; [Adzhubei, et al., 2010](#_ENREF_1) [[10](#_ENREF_10)] |
| MutationAssessor | MutationAssessor score predicts the functional impact of amino-acid substitutions in proteins based on evolutionary conservation of the affected amino acid in protein homologs. The score ranges from -5.2 to 6.5. | <http://mutationassessor.org/>; [Reva, et al., 2011](#_ENREF_14) [[11](#_ENREF_11)] |
| PROVEAN | PROVEAN predicts the functional effects of protein missense mutations by sequence conservation of the variant against a subset of clusters of sequences obtained by BLAST hits. Scores less than default threshold of -2.5 indicate deleterious variant, while scores above threshold indicate neutral variant. The score ranges from -14.0 to 14.0 with a smaller value representing more damaging effect. | <http://provean.jcvi.org/about.php>; [Choi, et al., 2012](#_ENREF_2) [[12](#_ENREF_12)] |
| FATHMM | FATHMM predicts the functional effects of protein missense mutations by combining homologous sequence conservation with domain intolerance scores. The score is the log odds of mutation versus wild-type model, or a similar log odds incorporating the variant’s associated domain intolerance score. The score ranges from -16.2 to 10.7 with a smaller value representing more damaging effect. | <http://fathmm.biocompute.org.uk/>; [Shihab, et al., 2013](#_ENREF_16) [[13](#_ENREF_13)] |

1. Grantham R (1974) Amino acid difference formula to help explain protein evolution. Science 185: 862-864. doi: 10.1126/science.185.4154.862

2. Cooper GM, Stone EA, Asimenos G, Green ED, Batzoglou S, et al. (2005) Distribution and intensity of constraint in mammalian genomic sequence. Genome Res 15: 901-913. doi: 10.1101/gr.3577405 PMID: 15965027

3. Siepel A, Bejerano G, Pedersen JS, Hinrichs AS, Hou M, et al. (2005) Evolutionarily conserved elements in vertebrate, insect, worm, and yeast genomes. Genome Res 15: 1034-1050. doi: 10.1101/gr.3715005 PMID: PMC1182216

4. Tavtigian SV, Deffenbaugh AM, Yin L, Judkins T, Scholl T, et al. (2006) Comprehensive statistical study of 452 BRCA1 missense substitutions with classification of eight recurrent substitutions as neutral. J Med Genet 43: 295-305. doi: 10.1136/jmg.2005.033878 PMID: 16014699

5. Kumar P, Henikoff S, Ng PC (2009) Predicting the effects of coding non-synonymous variants on protein function using the SIFT algorithm. Nat Protocols 4: 1073-1081. doi: 10.1038/nprot.2009.86

6. Li B, Krishnan VG, Mort ME, Xin F, Kamati KK, et al. (2009) Automated inference of molecular mechanisms of disease from amino acid substitutions. Bioinformatics 25: 2744-2750. doi: 10.1093/bioinformatics/btp528 PMID: 19734154

7. Garber M, Guttman M, Clamp M, Zody MC, Friedman N, et al. (2009) Identifying novel constrained elements by exploiting biased substitution patterns. Bioinformatics 25: i54-i62. doi: 10.1093/bioinformatics/btp190

8. Chun S, Fay JC (2009) Identification of deleterious mutations within three human genomes. Genome Res 19: 1553-1561. doi: 10.1101/gr.092619.109 PMID: 19602639

9. Pollard KS, Hubisz MJ, Rosenbloom KR, Siepel A (2010) Detection of nonneutral substitution rates on mammalian phylogenies. Genome Res 20: 110-121. doi: 10.1101/gr.097857.109 PMID: 19858363

10. Adzhubei IA, Schmidt S, Peshkin L, Ramensky VE, Gerasimova A, et al. (2010) A method and server for predicting damaging missense mutations. Nat Methods 7: 248-249. doi: 10.1038/nmeth0410-248 PMID: 20354512

11. Reva B, Antipin Y, Sander C (2011) Predicting the functional impact of protein mutations: application to cancer genomics. Nucleic Acids Res 39: e118. doi: 10.1093/nar/gkr407 PMID: 21727090

12. Choi Y, Sims GE, Murphy S, Miller JR, Chan AP (2012) Predicting the functional effect of amino acid substitutions and indels. PLoS One 7: e46688. doi: 10.1371/journal.pone.0046688 PMID: 23056405

13. Shihab HA, Gough J, Cooper DN, Stenson PD, Barker GL, et al. (2013) Predicting the functional, molecular, and phenotypic consequences of amino acid substitutions using hidden Markov models. Hum Mutat 34: 57-65. doi: 10.1002/humu.22225 PMID: 23033316
