# Supplementary material for: A Bayesian framework for efficient and accurate variant prediction
Source: PLoS One. 2018 Sep 13;13(9):e0203553. doi: 10.1371/journal.pone.0203553 (PMC6136750; doi:10.1371/journal.pone.0203553)
Supplement: S2 Table — a The 6 meta predictors in this table will be used to evaluate the prediction performance. (DOCX) [file pone.0203553.s002.docx]

**S2 Table. Meta *in silico* predictors**

| **Predictor^a^** | **Method Description** | **Website and Reference** |
| --- | --- | --- |
| MutationTaster | MutationTaster measures the effect of intronic and hard to interpret synonymous mutations by using a Bayes classifier to predict the disease potential of a variant. The classifier learns the probability that a variant is a deleterious mutation. The score ranges from 0 to 1. | <http://www.mutationtaster.org/>; Schwarz, et al., 2014 [[1](#_ENREF_1)] |
| CADD | CADD score integrates multiple annotations into one metric by contrasting variants that survived natural selection with simulated mutations. The score ranges from 0 to 99. | <http://cadd.gs.washington.edu/>; [Kircher, et al., 2014](#_ENREF_10) [[2](#_ENREF_2)] |
| REVEL | REVEL is an ensemble method for predicting the pathogenicity of missense variants based on a combination of scores from 13 individual tools: MutPred, FATHMM v2.3, VEST 3.0, Polyphen-2, SIFT, PROVEAN, MutationAssessor, MutationTaster, LRT, GERP++, SiPhy, phyloP, and phastCons. The score ranges from 0 to 1. | <https://sites.google.com/site/revelgenomics/>; [Ioannidis, et al., 2016](#_ENREF_7) [[3](#_ENREF_3)] |
| Eigen  Eigen PC | Eigen is an unsupervised learning algorithm that combines a wide range of predictors to form clusters of functional and non-functional nucleotide positions in the genome. The scores Eigen and Eigen_PC each ranges from -3 to 2. | [Ionita-Laza, et al., 2016](#_ENREF_8) [[4](#_ENREF_4)] |
| MetaSVM | MetaSVM is a support vector machine trained with high-throughput omic data. This is a gene-level classifier (feature reduction through lasso), useful for identifying genes associated with disease biomarkers such as expression levels. The score ranges from -2 to 3. | <https://omictools.com/meta-svm-tool>; [Kim, et al., 2017](#_ENREF_9) [[5](#_ENREF_5)] |

1. Schwarz JM, Cooper DN, Schuelke M, Seelow D (2014) MutationTaster2: mutation prediction for the deep-sequencing age. Nat Methods 11: 361-362. doi: 10.1038/nmeth.2890 PMID: 24681721

2. Kircher M, Witten DM, Jain P, O'Roak BJ, Cooper GM, et al. (2014) A general framework for estimating the relative pathogenicity of human genetic variants. Nat Genet 46: 310-315. doi: 10.1038/ng.2892 PMID: 24487276

3. Ioannidis NM, Rothstein JH, Pejaver V, Middha S, McDonnell SK, et al. (2016) REVEL: An ensemble method for predicting the pathogenicity of rare missense variants. Am J Hum Genet 99: 877-885. doi: 10.1016/j.ajhg.2016.08.016 PMID: 27666373

4. Ionita-Laza I, McCallum K, Xu B, Buxbaum JD (2016) A spectral approach integrating functional genomic annotations for coding and noncoding variants. Nat Genet 48: 214-220. doi: 10.1038/ng.3477 PMID: 26727659

5. Kim S, Jhong JH, Lee J, Koo JY (2017) Meta-analytic support vector machine for integrating multiple omics data. BioData Min 10: 2. doi: 10.1186/s13040-017-0126-8 PMID: 28149325
