# Supplementary material for: A Bayesian framework for efficient and accurate variant prediction
Source: PLoS One. 2018 Sep 13;13(9):e0203553. doi: 10.1371/journal.pone.0203553 (PMC6136750; doi:10.1371/journal.pone.0203553)
Supplement: S3 Table — a No. of variants in gene-specific training data included the classified variants in MGPT data from the same gene and those from other genes with minimal average distance to meet the sample size criterion of nneg ≥ 5 and npos ≥ 5. b The IVP models were built on a subset of 16 standardized scores (gra, …, mua) in unit range of 0 to 1 from their original scores (Grantham, …, FATHMM), as follows: gra = (Grantham– 5)/215, ger = (GERP++ + 12.3)/18.5, pcv = phastCons_vertebrate, pcm = phastCons_mannalian, agv = AGVGD/65, sif = 1 –SIFT, mup = MutPred, sip = Siphy/38, lrt = LRT, ppv = (phyloP_vertebrate + 20)/30, ppm = (phyloP_mammalian + 13.3)/14.5, pov = Polyphen2_HVAR, pod = Polyphen2_HDIV, mua = (MutationAssessor + 5.2)/11.7, pro = (PROVEAN + 14)/28, and fat = (FATHMM + 16.2)/26.9. (DOCX) [file pone.0203553.s003.docx]

**S3 Table. *In silico* predictors retained in gene-specific IVP models**

| **Gene** | **No. of Variants^a^** | **IVP Model^b^** |
| --- | --- | --- |
| *BRCA1* | 269 | logit(y) = -27.8353 + 21.9841 × ppv + 3.2853 × mup + 16.0141 × fat + 1.8049 × agv |
| *BRCA2* | 385 | logit(y) = -17.8681 + 9.4451 × ppv + 2.8744 × pcm + 9.2214 × mua |
| *CDH1* | 28 from *CDH1*; 2 from *BRCA1* | logit(y) = -2.0158 + 3.3374 × agv |
| *PALB2* | 22 from *PALB2*; 5 from *BRCA2* | logit(y) = -10.5538 + 27.3950 × sip |
| *PTEN* | 58 from *PTEN*; 5 from *MLH1* | logit(y) = 0.8902 + 3.3812 × agv |
| *TP53* | 103 | logit(y) = -1630.3455 + 2633.0005 × mua + 140.1740 × pcm |
| *MLH1* | 125 | logit(y) = -1.4591 + 3.4900 × pov |
| *MSH2* | 82 | logit(y) = -4.6816 + 2.3656 × agv + 3.4873 × gra + 3.5280 × sif |
| *MSH6* | 51 | logit(y) = -6.0865 + 8.3034 × pov |
| *PMS2* | 38 from *PMS2*; 2 from *BRCA2* | logit(y) = -25.6670 + 36.2577 × mua |
