# Supplementary material for: A Bayesian framework for efficient and accurate variant prediction
Source: PLoS One. 2018 Sep 13;13(9):e0203553. doi: 10.1371/journal.pone.0203553 (PMC6136750; doi:10.1371/journal.pone.0203553)
Supplement: S4 Table — a IVP model analysis was conducted with 1,161 missense variants. MVP model analyses were evaluated in 1,016 variants with any available evidence and 873 variants with only auto-computed evidence. Total numbers of variants for MVP model analysis were reduced due to some variants did not have the required evidence statistics. Known classes were based on ClinVar consensus classification outcomes, and the predicted classes were evaluated by IVP and MVP models, respectively, using LOOCV. (DOCX) [file pone.0203553.s004.docx]

**S4 Table. Cross validation for variant classification in MGPT data**

|  | **Number (Row %) by Predicted Classes** | | | | |
| --- | --- | --- | --- | --- | --- |
| **Known Class by Method^a^** | **Benign** | **VLB** | **VUS** | **VLP** | **Pathogenic** |
| IVP model analysis |  |  |  |  |  |
| Benign (n = 267) | 3 (1.1%) | 176 (65.9%) | 88 (33.0%) | 0 | 0 |
| VLB (n = 480) | 11 (2.3%) | 299 (62.3%) | 170 (35.4%) | 0 | 0 |
| VLP (n = 199) | 0 | 1 (0.5%) | 124 (62.3%) | 23 (11.6%) | 51 (25.6%) |
| Pathogenic (n = 215) | 0 | 3 (1.4%) | 138 (64.2%) | 39 (18.1%) | 35 (16.3%) |
| Total (n = 1,161) | 14 (1.2%) | 479 (41.3%) | 520 (44.8%) | 62 (5.3%) | 86 (7.4%) |
|  |  |  |  |  |  |
| MVP model analysis using all available evidence |  |  |  |  |  |
| Benign (n = 243) | 131 (53.9%) | 43 (17.7%) | 67 (27.6%) | 2 (0.8%) | 0 |
| VLB (n = 443) | 67 (15.1%) | 278 (62.8%) | 98 (22.1%) | 0 | 0 |
| VLP (n = 156) | 0 | 0 | 38 (24.4%) | 60 (38.5%) | 58 (37.2%) |
| Pathogenic (n = 174) | 0 | 0 | 29 (16.7%) | 63 (36.2%) | 82 (47.1%) |
| Total (n = 1,016) | 198 (19.5%) | 321 (31.6%) | 232 (22.8%) | 125 (12.3%) | 140 (13.8%) |
|  |  |  |  |  |  |
| MVP model analysis using only auto-computed evidence |  |  |  |  |  |
| Benign (n = 241) | 126 (52.3%) | 39 (16.2%) | 74 (30.7%) | 1 (0.4%) | 1 (0.4%) |
| VLB (n = 377) | 35 (9.3%) | 87 (23.1%) | 255 (67.6%) | 0 | 0 |
| VLP (n = 120) | 0 | 0 | 83 (69.2%) | 32 (26.7%) | 5 (4.2%) |
| Pathogenic (n = 135) | 0 | 0 | 77 (57.0%) | 43 (31.9%) | 15 (11.1%) |
| Total (n = 873) | 161 (18.4%) | 126 (14.4%) | 489 (56.0%) | 76 (8.7%) | 21 (2.4%) |
