# Supplementary material for: A Bayesian framework for efficient and accurate variant prediction
Source: PLoS One. 2018 Sep 13;13(9):e0203553. doi: 10.1371/journal.pone.0203553 (PMC6136750; doi:10.1371/journal.pone.0203553)
Supplement: S5 Table — (DOCX) [file pone.0203553.s005.docx]

**S5 Table. Number of variants according to ClinVar consensus classification**

| **Gene by Dataset** | **Benign** | **VLB** | **VLP** | **Pathogenic** | **Total** |
| --- | --- | --- | --- | --- | --- |
| All variants: |  |  |  |  |  |
| *BRCA1* | 100 | 106 | 23 | 40 | 269 |
| *BRCA2* | 107 | 239 | 17 | 22 | 385 |
| *CDH1* | 3 | 20 | 5 | 0 | 28 |
| *PALB2* | 13 | 9 | 0 | 0 | 22 |
| *PTEN* | 0 | 0 | 25 | 33 | 58 |
| *TP53* | 2 | 13 | 52 | 36 | 103 |
| *MLH1* | 11 | 25 | 36 | 53 | 125 |
| *MSH2* | 7 | 23 | 25 | 27 | 82 |
| *MSH6* | 5 | 30 | 13 | 3 | 51 |
| *PMS2* | 19 | 15 | 3 | 1 | 38 |
| Total | 267 | 480 | 199 | 215 | 1,161 |
|  |  |  |  |  |  |
| Variants with any evidence statistics: |  |  |  |  |  |
| *BRCA1* | 86 | 92 | 20 | 36 | 234 |
| *BRCA2* | 102 | 225 | 15 | 19 | 361 |
| *CDH1* | 3 | 19 | 5 | 0 | 27 |
| *PALB2* | 13 | 9 | 0 | 0 | 22 |
| *PTEN* | 0 | 0 | 16 | 24 | 40 |
| *TP53* | 2 | 13 | 49 | 33 | 97 |
| *MLH1* | 9 | 23 | 24 | 37 | 93 |
| *MSH2* | 6 | 22 | 12 | 22 | 62 |
| *MSH6* | 5 | 27 | 12 | 2 | 46 |
| *PMS2* | 17 | 13 | 3 | 1 | 34 |
| Total | 243 | 443 | 156 | 174 | 1,016 |
|  |  |  |  |  |  |
| Variants with any of the 3 auto-computed evidence statistics: |  |  |  |  |  |
| *BRCA1* | 85 | 75 | 17 | 28 | 205 |
| *BRCA2* | 101 | 196 | 11 | 17 | 325 |
| *CDH1* | 3 | 15 | 4 | 0 | 22 |
| *PALB2* | 13 | 7 | 0 | 0 | 20 |
| *PTEN* | 0 | 0 | 13 | 22 | 35 |
| *TP53* | 2 | 9 | 41 | 27 | 79 |
| *MLH1* | 9 | 22 | 16 | 29 | 76 |
| *MSH2* | 6 | 16 | 8 | 11 | 41 |
| *MSH6* | 5 | 25 | 7 | 0 | 37 |
| *PMS2* | 17 | 12 | 3 | 1 | 33 |
| Total | 241 | 377 | 120 | 135 | 873 |
