# Supplementary material for: A Bayesian framework for efficient and accurate variant prediction
Source: PLoS One. 2018 Sep 13;13(9):e0203553. doi: 10.1371/journal.pone.0203553 (PMC6136750; doi:10.1371/journal.pone.0203553)
Supplement: S6 Table — a The description of each evidence item follows the Ambry's Variant Classification Scheme. b All evidence items are summarized into 7 categories for MVP model analysis: FAA: Frequency and association evidence from control populations and case-control studies; COC: Co-occurrence evidence with another mutation; CSG: Co-segregation evidence with disease; FHX: Evidence of personal and family history, de novo alternation in family and established diagnosis without other mutation; FUN: Evidence of functional validation or genomic features (includes mutational hotspot); STR: Structural evidence; OTH: Other supporting evidence not included in other evidence groups. c The effect of each evidence item is graded into 7 levels of P-1, P-4, LP-1, LP-4, LB-1, LB-2 and B-1, respectively. These effect levels are quantified using following criteria under the assumption of no prior knowledge of variant pathogenicity: One evidence item of effect level P-1, or 4 evidence items of effect level P-4, are required to classify a variant to pathogenic; One evidence item of effect level LP-1, or 4 evidence items of effect level LP-4, are required to classify a variant to VLP; One evidence item of effect level LB-1, or 2 evidence items of effect level LB-2, are required to classify a variant to VLB; One evidence item of effect level B-1 is required to classify a variant to benign. d The effect of each qualitative evidence is transferred to a LR statistic under a null probability of 0.5 (see Eq (1) in Methods section). Numerically, the LR statistics for different evidence effects are calculated as follows: LR(P-1) = 0.99/(1–0.99) = 99; LR(P-4) = 990.25 = 3.1543; LR(LP-1) = 0.95/(1–0.95) = 19; LR(LP-4) = 190.25 = 2.0878; LR(LB-1) = 0.05/(1–0.05) = 0.0526; LR(LB-2) = (1/19)0.5 = 0.2294; LR(B-1) = 0.001/(1–0.001) = 0.0010. (DOCX) [file pone.0203553.s006.docx]

**S6 Table. Evidence items and their likelihood ratio statistics**

| **Targeted**  **Class** | **Description of Evidence Item^a^** | **Evidence Group^b^** | **Evidence Effect^c^** | **LR Statistic^d^** |
| --- | --- | --- | --- | --- |
| Pathogenic | Confirmed *de novo* alteration in the setting of a new disease (appropriate phenotype) in the family | FHX | P-1 | 99 |
|  | Alterations resulting in premature truncation (e.g. reading frame shift, nonsense) | FUN |  |  |
|  | Other ACMG-defined mutation (i.e. initiation codon or gross deletion) | OTH |  |  |
|  | Strong segregation with disease (LOD >3 or >10 meioses) | CSG |  |  |
|  | Functionally-validated splicing mutation | FUN |  |  |
|  | Significant disease association in appropriately sized case-control study(ies) | FAA | P-4 | 3.1543 |
|  | Detected in individual satisfying established diagnostic criteria for classic disease without a clear mutation | FHX |  |  |
|  | Last nucleotide of exon | FUN |  |  |
|  | Good segregation with disease (LOD 1.5-3 or 5-9 meioses) | CSG |  |  |
|  | Deficient protein function in appropriate functional assay(s) | FUN |  |  |
|  | Well-characterized mutation at same position | FUN |  |  |
|  | Other strong data supporting pathogenic classification | OTH |  |  |
|  | Structural evidence | STR |  |  |
| VLP | Alterations at the canonical donor/acceptor sites (+/- 1, 2) without other strong (b-level) evidence supporting pathogenicity | FUN | LP-1 | 19 |
|  | Rarity in general population databases (ESP, 1000 genomes, ExAC, gnomAD) | FAA | LP-4 | 2.0878 |
|  | Moderate segregation with disease (at least 3 informative meioses) for rare diseases | CSG |  |  |
|  | Other data supporting pathogenic classification | OTH |  |  |
|  | Structural evidence | STR |  |  |
| VLB | Intact protein function observed in appropriate functional assay(s) | FUN | LB-1 | 0.0526 |
|  | Intronic alteration with no splicing impact by RT-PCR analysis or other splicing assay | FUN |  |  |
|  | Synonymous alterations with insufficient evidence to classify as benign | FUN |  |  |
|  | Other strong data supporting benign classification | OTH |  |  |
|  | Co-occurrence with mutations in same gene (phase unknown) | COC | LB-2 | 0.2294 |
|  | Co-occurrence with mutations in another gene that clearly explains a proband's phenotype | COC |  |  |
|  | Subpopulation frequency in support of benign classification | FAA |  |  |
|  | Does not segregate with disease in family study (genes with incomplete penetrance) | CSG |  |  |
|  | No disease association in small case-control study | FAA |  |  |
|  | Other data supporting benign classification | OTH |  |  |
|  | Structural evidence | STR |  |  |
| Benign | General population or subpopulation frequency is too high to be a pathogenic mutation based on disease/syndrome prevalence and penetrance | FAA | B-1 | 0.0010 |
|  | Does not segregate with disease in family study (genes with complete penetrance) | CSG |  |  |
|  | Internal frequency is too high to be a pathogenic mutation based on disease/syndrome prevalence and penetrance | FAA |  |  |
|  | Seen in trans with a mutation or in homozygous state in individual without severe disease for that gene | COC |  |  |
|  | No disease association in appropriately sized case-control study(ies) | FAA |  |  |
