# Supplementary material for: A Bayesian framework for efficient and accurate variant prediction
Source: PLoS One. 2018 Sep 13;13(9):e0203553. doi: 10.1371/journal.pone.0203553 (PMC6136750; doi:10.1371/journal.pone.0203553)
Supplement: S7 Table — a The number of “total positive variants evaluated” includes true positives (TP), false negatives (FN) and those positives predicted as variants of uncertain significance (VUS). The number of “total negative variants evaluated” includes true negatives (TN), false positives (FP) and those negatives predicted as VUS. (DOCX) [file pone.0203553.s007.docx]

**S7 Table. Performance statistics for variant classification**

| **Performance Statistic (Abbreviation)** | **Formula or Definition^a^** |
| --- | --- |
| Sensitivity (Sen; Also called true positive rate) | $Sen=\frac{TP}{Total positive variants evaluated}$ |
| Specificity (Spe; Also called 1 minus false positive rate) | $Spe=\frac{TN}{Total negative variants evaluated}$ |
| Positive predictive value (PPV) | $PPV=\frac{TP}{TP+FP}$ |
| Negative predictive value (NPV) | $NPV=\frac{TN}{TN+FN}$ |
| Accuracy (Acc) | $Acc=\frac{TP+TN}{TP+TN+FP+FN}$ |
| Area under the receiver operating characteristic curve (AUC) | The AUC value is quantified using trapezoidal rule in variants predicted as benign, VLB, VLP and pathogenic. |
| Proportion of variants of uncertain significance (P_VUS_) | $P_{VUS}=\frac{No. of predicted VUSs}{Total variants evaluated}$ |
